# Fearfulness Affects Quail Maternal Care and Subsequent Offspring Development

**DOI:** 10.1371/journal.pone.0102800

**Published:** 2014-07-17

**Authors:** Florent Pittet, Cécilia Houdelier, Océane Le Bot, Christine Leterrier, Sophie Lumineau

**Affiliations:** 1 Université Rennes 1, Centre National de la Recherche Scientifique, Unité Mixte de Recherche 6552 « Ethos », Rennes, France; 2 Institut National de la Recharche Agronomique, Unité mixte de Recherche 85 Physiologie de la Reproduction et des Comportements, Nouzilly, France; University of Rouen, France, France

## Abstract

Our study investigated relationships between a precocial bird’s fearfulness and maternal care, and the implication of maternal care as a vector for non-genomic transmission of fearfulness to chicks. We compared care given to chicks between two sets of female Japanese quail selected to present either high (LTI) or low fearfulness (STI). Chicks, from a broiler line, were adopted by these females following a sensitization procedure. Chicks’ fearfulness after separation from their mother was assessed by well-established procedures. LTIs took longer to present maternal responses, pecked chicks more during the first days post-hatch, presented impaired maternal vocal behaviour and were globally less active than STI females. Chicks mothered by LTIs presented more fearful reactions than did chicks mothered by STIs, supporting the hypothesis of a non-genetic maternal transmission of fearfulness. We suggest that the longer latencies required by LTIs to become maternal are a consequence of their greater fear of chicks, and that their lower general and vocal activity could be components of a heightened antipredatory strategy. We discuss the transmission of maternal fearfulness to fostered chicks, taking into account the possible implication of several well-known mechanisms underlying maternal effects.

## Introduction

Early rearing environment, the main constituent of which is the mother for a large range of species, strongly impacts the behavioural development of individuals [1]–[3]. Maternal effects due to non-genomic transmission of behavioural characteristics from mother to offspring have been described. Cases of social heredity in mammals concern in particular consistent individual differences in both maternal behaviour (i.e. maternal styles) [4]–[6] and non-reproductive behaviour (i.e. temperament traits or personalities) [7], [8]. As these individual behavioural characteristics partly result from each female’s experience, their transmission to offspring cannot be ensured by genetic mechanisms alone. To enable this “social heredity”, maternal care has been identified as a bridge between mammalian mothers’ and their offspring’s behavioural characteristics.

Reports illustrate the implication of mammals’ maternal care in non-genomic transmission of fearfulness. Fearfulness is a multidimensional temperament trait [9] that can be defined as a psychological profile resulting in an individual’s consistent reactivity to fear-eliciting situations. Fear responses are critical for survival in natural situations as they allow individuals to escape from predators and other dangers [10]. This temperament dimension can have repercussions on cognitive skills [11] and on many essential behaviours [12] including exploratory behaviour [13], [14] or social interactions [15]. Mammalian females’ fearfulness affects the expression of their maternal care [16]–[18] and, in turn, their care characteristics can influence the development of their offspring’s fearfulness [2], [4]. These results indicate that maternal care characteristics ensure a link between maternal fearfulness and offspring fearfulness, as demonstrated for altricial rodent females from strains presenting large differences in fearfulness [7]. Nevertheless, mammal mothers not only influence their offspring via their maternal behaviour but also via physiological signals conveyed by milk. For instance, rodents’ milk glucocorticoid concentrations depend on maternal circulating glucocorticoids and have long-term consequences on offspring behaviour and cognition, see [19] for a review. Thus, even in the rare cases of cross-fostering procedures, mammals never offer the opportunity to disentangle maternal behavioural from physiological influences, making research on alternative biological models such as birds particularly interesting.

Precocial birds’ maternal effects are known to be particularly strong. The ease to perform total maternal deprivation procedures without significant human intervention enabled researchers to illustrate impairment of the behaviour of motherless offspring [3], [20]–[23]. Furthermore, precocial birds’ maternal effects have been illustrated by cases of non-genomic transmission of maternal temperament to fostered chicks, particularly concerning fearfulness: chicks fostered by fearful females behave, after separation from their mothers, more fearfully than chicks brooded by females with low fearfulness [24], [25]. Contrary to mammals, behavioural mechanisms involved in non-genomic transmission of fearfulness remain unidentified in bird species. However, precocial birds are becoming choice models to study maternal effects. Indeed, adult females of many precocial bird species express spontaneously a complete and rich maternal behaviour repertoire without humans having to intervene [26], [27]. In addition, the absence of lactation enables the evaluation of a purely behavioural influence of mother on offspring. Recent reports described the existence of Japanese quail’s maternal styles, i.e. consistent individual differences in care behaviours [27]. Maternal styles can be predicted by maternal temperament and these styles affected chicks’ behavioural development. Pittet et al. (2014) demonstrated that several behaviours related to the “rejection” dimension of care were correlated with both mothers’ and chicks’ social motivations. This demonstrates the implication of this care dimension in the transmission of mothers’ social characteristics to their chicks. The other dimension of care, labelled “aggression”, was correlated with maternal fearfulness, but the authors could not demonstrate correlations between mothers’ aggression scores and chicks’ fearfulness. Pittet et al. (2014) proposed that this last result could be due to insufficient individual differences between mothers’ fearfulness (and consequently aggressive styles).

The present study investigates how fearfulness affects the expression of maternal care and how this maternal care is involved in non-genomic transmission of fearfulness in precocial birds. The biological model for this investigation was Japanese quail (*Coturnix c. japonica*), a precocial bird species the females of which are the only care-givers. Through artificial selection, two well-established lines characterized either by high or by low fearfulness (respectively LTIs end STIs) have been obtained [28]. Experimenting with females from these two lines, we evaluated how fearfulness influenced maternal responses by comparing maternal care expressed by LTIs and STIs during fostering procedures. We then compared the behavioural characteristics of chicks brooded by these two lines of females to determine how potential differences in maternal care affected their behavioural development. We hypothesized that care characteristics would differ according to the fearfulness of hens and that fearfulness would be socially transmitted to chicks. We also hypothesized that correlational data would highlight a link between maternal care behaviours related to the aggressiveness dimension of styles and chicks’ fearfulness.

## Methods

### Ethic statement

All experiments were approved by the departmental direction of veterinary services (Ille-et-Vilaine, France, permit number 005283) and were performed in accordance with the European Communities Council Directive of 24 November 1986 (86/609/EEC). Our brooding procedure and our ethological tests were approved by the regional ethics committee (agreement number: R-2011-SLU-02).

### Subjects and rearing conditions

Forty-four 8 month-old female Japanese quail of the 45th generation of two divergent lines selected for different levels of fearfulness on the basis of their tonic immobility duration were given 176 chicks to adopt. Both divergent lines are produced and maintained at the INRA experimental unit 1295 (UE PEAT, F-37380 Nouzilly, France). Individuals have been selected on the basis of their tonic immobility duration (TI), a natural antipredatory reaction characterized by a catatonic state of the animal, whose duration is a good index of inherent fearfulness [28]. Tonic immobility can be induced easily by placing a bird on its back and by restraining it in this position for a few seconds prior to release. The subject then remains immobile for a various length of time and tonic immobility duration is measured by the time before it stands up. This test is non-invasive, well established, standardized and easy to perfrom [10]. Selection led to a line of quail presenting a long TI durations (LTI) and consequently high fearfulness, and a line presenting short TI durations (STI) and characterized by low fearfulness. Interestingly, this selection on TI duration not only modified TI response characteristics but also general fearfulness, as LTIs express more freezing in open-fields, emerge later in emergence tests [24], [29], take longer to approach a novel object [30] and are more reactive to humans [24] than STIs. This selection was accomplished independently of social reinstatement tendencies [31].

Adult females were individualized by a numbered ring on a wing when they arrived at the laboratory and were placed individually in wire mesh brooding cages (51×40×35 cm) with a drinker and a feeder. The light/dark cycle was 12/12 and the room temperature 20±1°C. Females were weighed when they arrived at the laboratory, the day they adopted chicks and the day they were separated from chicks.

Adopted chicks of a broiler line were used. They came from eggs provided by an industrial farm (Les cailles de Chanteloup, Corps-Nuds, France) and were artificially incubated in our laboratory. Incubation lasted 17 days (37.7°C, 45–50% humidity). After hatching, chicks were placed in groups of 40 in large plastic cages (98×35×42 cm) equipped with a feeder, a water source and a heater (38±1°C). The evening on the day chicks hatched they were divided into two sets and each set was subdivided into 22 group of 4 chicks. Each group of 4 chicks was placed with either a LTI or a STI mother. Chicks fostered by LTIs are named LTI-cs and chicks fostered by STIs are named STI-cs. As morphological sexual dimorphism appears only 3 weeks after hatching [32], male and female chicks were randomly distributed to each set and their sex was determined when they were 3 weeks old. Sex ratios of chicks did not differ between sets (% males: LTI: 47.1%, STI: 43.4%; χ^2^_1_ = 0.24, p = 0.6). Chicks were weighed when they were 11 days old and 18 days old.

### Maternal induction and brooding procedure

Three weeks before brooding was induced, 22 LTIs and 22 STIs were placed in brooding cages to habituate to their environment. They were distributed so that two females of the same set were never in neighbouring cages. During this habituation period, the TI responses of LTIs and STIs were evaluated to check differences resulting from selection.

The day following hatching, when the light was switched off (08∶00 pm) chicks were placed by groups of 4 underneath adult females that had been enclosed an hour earlier in a nest box (18×18×18 cm) and locked-up for the night during which the chicks’ vocal and physical solicitations induced rapid expression of maternal behaviour by the adult females. Details of this procedure are described by Richard-Yris et al. [26]. The next morning, all the boxes were opened and removed from the cages. Chicks that showed signs of hypothermia (motionless, trembling, eyes closed, difficulties to emit distress calls) when leaving the boxes were replaced by chicks that were not tested subsequently. At this stage, the females that did not express any chick warming behaviour were excluded from the experience: one STI but no LTI had to be excluded. Chicks that showed signs of hypothermia and did not stimulate their mother were replaced by chicks of same age so that all broods had the same number of chicks (4). Replacement chicks were identified by a colour leg-ring, and were not tested after the brooding period. The number of replacements did not differ significantly between LTIs and STIs broods (LTI: 9/88, STI: 6/84, χ^2^_1_ = 0.51, p = 0.5).

During the 11 days that brooding lasts naturally [33], we recorded interactions between hens and chicks; details of observations of maternal behaviour are described below. Hens were removed when chicks were 11 days old. Chicks then developed in sibling groups for two more weeks during which the fearfulness of two chicks chosen randomly in each cage was evaluated by several ethological tests. Sex-ratio of tested chicks did not differ significantly between the sets (%males: LTI: 40.5%; STI: 47.6%; χ^2^_1_ = 0.43, p = 0.5).

### Characterization of maternal behaviour

Observations of maternal behaviour were all performed in the brooding room, behind one-way mirrors and the observer was blind toward the animal’s sets. Observations followed the procedure previously used to characterize maternal care in Japanese quail [34], [35].

#### Response to sensitization

During the first half-day that the mothers spent with chicks, their maternal behaviour was recorded by instantaneous scan sampling at 5-minute intervals during the first two hours and at 15-minute intervals during the following two hours. Each scan recorded how many chicks showed signs of hypothermia, whether the mother was warming the chicks and whether she cooed, a typical maternal vocalization. This early observation enabled us to determine the speed of emergence of maternal responses.

#### Observations of maternal care

Maternal behaviour was recorded on post-hatch day 2 (PHD2), PHD3, PHD5, PHD7 and PHD9. We assessed maternal behaviour using both instantaneous scan samplings to establish mothers’ time-budgets, associated with focal animal sampling to note rare behaviours. The observer was hidden behind a one-way mirror.

#### Instantaneous scan sampling

Each day we recorded 60 scans at 5-minute intervals: 30 scans in the morning and 30 in the afternoon. Each scan recorded whether the mother was warming chicks and, if she was, we recorded her posture (table 1). The mother’s activity was noted as well as the number of chicks at each distance class from the mother (table 1). This last measure enabled us to calculate an index for the mean distance between non-warmed chicks and mother. The formula weights distance classes to give more weight to the farthest distances and less weight to the nearest ones:

**Table 1:** Behavioural items recorded for LTI and STI females.

Measures		Definitions
**Warming** **activity**	Yes/no	Mother is motionless and at least one chick is partially or entirely covered by her feathers
**Warming** **posture**	Covering posture: Chick(s) is/arecompletely hidden under theirmother’s feathers	Lying down: Both feet and tibio-tarsal articulations touch the floor; body and neck hunched up, touching the floor
		Crouched: Both feet and tibio-tarsal articulations touch the floor, body is slightly raised, head raised up, feathers touch the floor but the belly does not
		Medium: Feet touch the floor, but tibio-tarsal articulations do not and feathers are close to the floor
	Non-covering posture: Chicks arepartially exposed to the environment	Lying on one side: The female is stretched out, her flank touches the floor, chicks must snuggle against her to be warmed
		High: The female is standing up, legs straight, her body is too high for the chicks to be completely covered
**Maternal** **activity**	Rest/observe/feed/explore/self-preen/dust bathe/jump/alert/peck chick/aggress chick	
**Distance** **chick-mother**	Under	Chick is under the female
	Close	Chick is not under the female but in contact with her
	Near	Chick is one chick length max from the female
	Far	Chick is between one chick length and half the cage away from the female
	Far away	Chick is between half the cage length and cage length
	Opposite	Mother is against one cage wall and chick is against the opposite wall



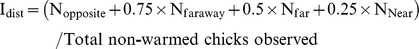
 (N: number of non-warmed chicks, subscript: distance class).

The behavioural traits recorded are described in table 1. Data were sampled using an ipod Touch (Apple) and the application “scan sampling” (Vincent Richard).

##### Focal sampling

Each cage was observed for two 4-minute sessions, one in the morning and one in the afternoon when mother’s entire behavioural sequence (occurrence of all maternal and non-maternal behaviours) was recorded. Additional traits were also noted: every warming break between each chick and its mother, including who initiated the break (the mother, by moving while warming, or the chick by moving away from its mother’s feathers) and trampling of chicks.

#### Separation test

When the chicks were 10 days old (PHD10), they were taken out of their cage away from their mother and the reactions of each mother were recorded during a 5-minute focal sampling. These data yielded the latency and frequencies of distress calls, of comfort behaviours such as resting or eating and the frequencies of all other behaviours.

### Evaluation of adult females’ and fostered chicks’ fearfulness

Mothers’ tonic immobility durations were evaluated during their habituation to their brooding environment. Chicks were tested after separation from their mothers, and we assessed their tonic immobility durations, their “shyness” in the emergence test, their behaviour in an open-field and their reaction to a sudden startling loud sound.

#### Tonic immobility test

This test followed the protocol described by Jones [36]. Tonic immobility (TI) is a reflexive response to a fear-inducing stimulus and response duration is positively correlated with fearfulness. Mothers were tested 2 weeks before the beginning of the brooding period and chicks were tested when they were 15 days old. Each test individual was removed from its cage and placed on its back in a U-shaped wooden cradle and held in this position for 10 seconds prior to release. The experimenter, placed out of the subject’s sight, recorded both number of induction(s) required to obtain a TI lasting at least 10 seconds, after a maximum of 5 inductions, and the duration of tonic immobility, with a maximum of 300 s. Zero second was scored when the subject never remained in TI duration for longer than 10 seconds.

#### Emergence test

Chicks were tested when they were 16 days old. Each test subject was removed from its home cage and transported in the dark, in a wooden box (18×18×18 cm). This box was then placed on the left side of the apparatus: a large and well-lit wooden box (62×60×33 cm) with wood-shavings covering the floor and an observation window. When the transport box was placed in the apparatus, it was kept closed for 1 minute and the latency of the first distress call and the numbers of calls emitted by the chick were recorded. Then, the door was left opened for 3 minutes. Latencies to pass its head out of the box and to emerge completely were recorded. In this test, the time taken by individuals to emerge from a shelter into an unknown environment is a good estimate of fearfulness [37]–[39]. Once the animal is in the test cage, the transport box is closed and the chick is observed for 3 minutes. The latency of its first distress call, the number of distress calls and the frequency of exploration, observation, locomotion and maintenance activities (grooming) were recorded.

#### Open-field test and response to a startling stimulus

Chicks were tested when they were 22–23 days old. Similar proportions of LTI-c and STI-c were tested each day. Chicks were placed individually in the dark in the centre of an arena (Ø120×60 cm) with white plastic walls and a linoleum floor. The experiment started when the light was switched on, and, hidden behind a one-way mirror, the experimenter recorded latency of first distress call, the number of distress calls, latency of first step, number of steps and frequency of observation, exploration and maintenance activities for 2 minutes. Then a short loud sound was broadcast. The immediate reaction of the subject and its behavioural expressions during the following two minutes were recorded.

### Statistical analyses

As most of the data were not normally distributed, we used non-parametric statistical tests to compare behavioural expressions between LTIs and STIs and their respective chicks. Mean frequencies (expressed in numbers per minute), latencies and proportions of scans of each set were compared using Mann-Witney U tests. Proportions of animals of each set that expressed or did not express a given behavioural trait were compared using Chi-square tests or Fisher exact probability tests for small samples. Correlation between maternal care and chicks’ fearfulness was estimated to determine the involvement of several mechanisms in the social transmission of fearfulness. To investigate the relationships between maternal styles and chicks’ behaviour, maternal variables were averaged from PHD2 to PHD5 following the method used to identify maternal styles [27]. We also tested the involvement of maternal behaviours that differed significantly between LTIs and STIs in the development of chicks’ fearfulness including latency to express warming, cooing from PHD2 to PHD5 and rejection on PHD9. To limit the number of correlation tests and thus avoid type I errors, these variables were only tested for their correlation with variables showing significant differences between chick sets. The relationship was tested using Spearman correlations between care behaviours of the mothers and the mean behaviour of their two chicks tested. The level of significance for all the tests was set at 0.05. Data analyses were computed using Statistica and XLStat.

## Results

### Mothers’ weights and fearfulness

LTIs’ TI durations were longer than STIs’ (TI duration: LTI: 245.82±17.43 s, STI: 20.32±2.61 s; Mann-Whitney U test: U = 8.5, p<0.0001) and the number of inductions failed were higher than those of (LTI: 0.09±0.06, STI: 2.24±0.43; U = 76, p<0.0001) revealing differences in emotionality.

Weights did not differ significantly among mothers either when they arrived at the laboratory (LTI: 190.91±3.27 g, STI: 197.08±4.20 g; U = 176, p = 0.2), or when they were given chicks (LTI: 212.20±6.06 g, STI: 202.18±6.48 g; U = 210, p = 0.6), or after separation from the chicks (LTI: 216.38±6.03 g, STI: 205.41±5.77 g; U = 284, p = 0.2).

### Maternal behaviour

#### Latency to express maternal behaviour

The morning following the induction procedure, we observed several differences between LTIs’ and STIs’ first maternal responses. LTIs’ first brooding behaviour took twice as long to appear as STIs’ (LTI: 45.0±7.1 min, STI: 23.8±1.3min.; U = 328.5, p = 0.01), and the average number of chicks showing signs of hypothermia (trembling, closed-eyes) was higher in LTIs’ than in STIs’ cages during that morning (LTI: 0.87±0.23, STI: 0.10±0.05; U = 110.5, p = 0.003). None of the 22 LTIs cooed whereas 13 STIs emitted this maternal call (Fisher exact probability test, p<0.001).

#### Maternal expressions during the brooding period

##### Warming behaviour

**Figure 1:**
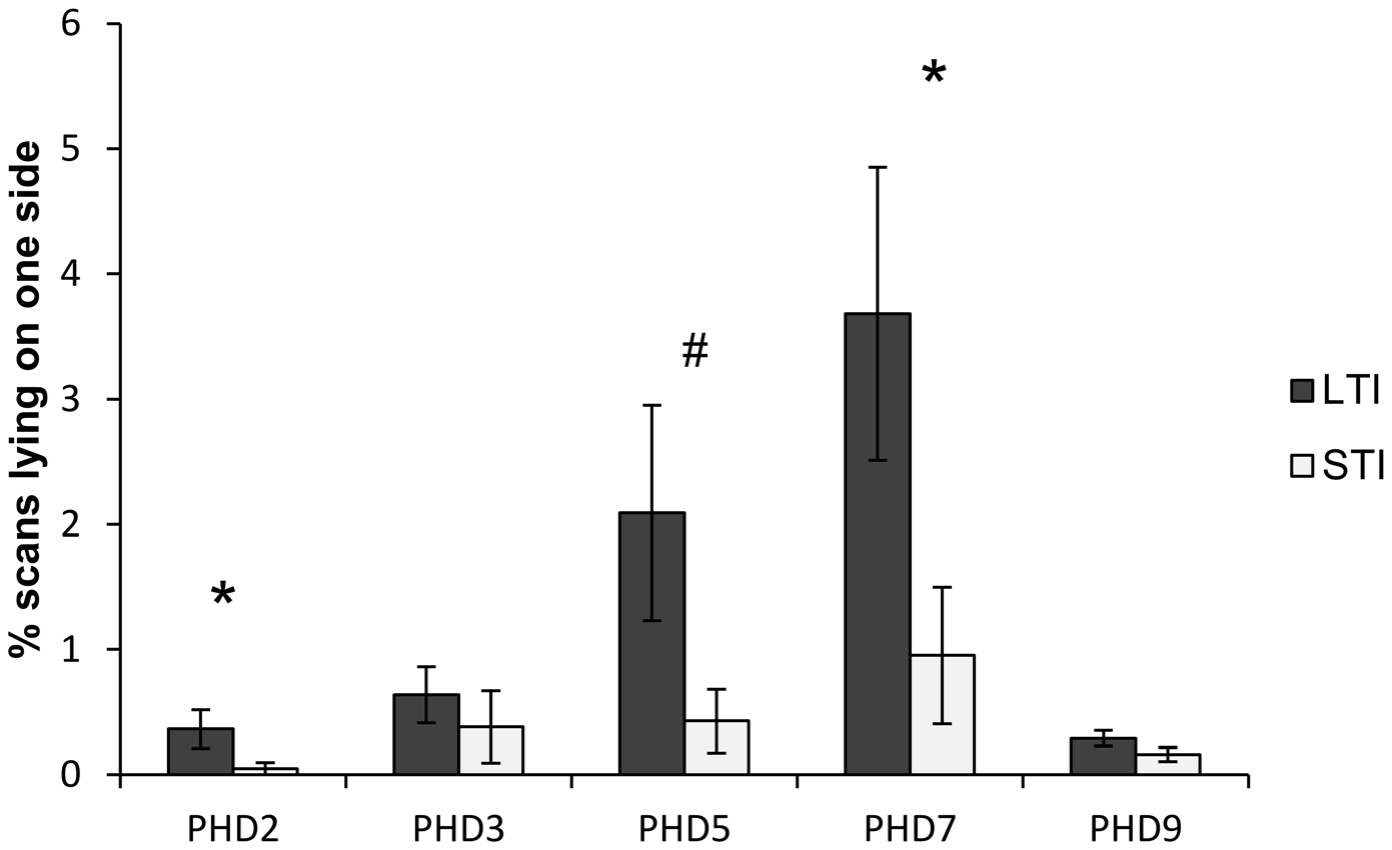
« Lying-on-one-side » by LTIs and STIs (% ± SEM). Mann-Whitney U-test *p<0.05, #p<0.08.

Frequencies of their different warming postures differed significantly between LTIs and STIs. When they were warming chicks, LTIs spent less time in covering postures on PHD7 and PHD9 (table 2). The proportion of warming time spent by LTIs by lying on one side was also higher on PHD2 (Mann-Whitney U test: U = 178.5, p = 0.05), PHD7 (U = 138, p = 0.015) and tended to be higher on PHD5 (U = 173.5, p = 0.09, Figure 1). Frequencies of warming breaks also differed, particularly on the last days of the brooding period. The mean frequency of warming breaks was higher in STIs’ broods on PHD7 (LTI: 0.288±0.049, STI: 0.393±0.042 per minute; U = 137.5, p = 0.05). On PHD9 the proportions of warming breaks initiated by mothers were higher for STIs’ broods, while those initiated by chicks were higher for LTIs’ broods (table 2). The times spent brooding chicks did not differ significantly between LTIs and STIs at any observed day (p>0.05).

**Table 2:** LTIs’ and STIs’ maternal care in relation to brooding day.

	PHD2		PHD3		PHD5		PHD7		PHD9	
Measures	LTI	STI	LTI	STI	LTI	STI	LTI	STI	LTI	STI
Warming(%scans)	73.2±5.3	80.3±1.4	84.3±1.5	84.0±1.4	72.3±2.4	71.0±2.9	62.7±4.0	61.6±4.3	40.2±4.7	37.7±5.1
Covering(% scans warming)	94.9±2.3	98.8±0.7	96.7±1.3	97.9±1.0	90.0±2.6	95.0±2.3	84.6±3.2*	**92.2±3.3**	62.6±5.9*	**77.2±5.7**
Warming break(% initiated by the mother)	47.0±14.4	43.1±11.1	51.2±9.4	54.0±9.1	55.1±8.5	47.4±8.3	55.3±9.3	42.4±8.6	24.2±10.4*	**59.9±11.1**
Cooing(frequency per minute)	0.682±0.473**	**5.250±2.220**	0.267±0.267*	**0.518±0.207**	0.057±0.039	0.190±0.155	0.210±0.013	–	–	–
Pecking(frequency per minute)	0.023±0.016	0.050±0.039	0.063±0.20*	0.006±0.006	**0.040±0.013#**	0.012±0.008	0.023±0.013	0.042±0.013	0.023±0.018	0.024±0.019
Trampling(frequency per minute)	0.006±0.006	0.019±0.014	0.079±0.040	0.190±0.096	–	0.036±0.025	–	–	–	–
Distance(index)	0.36±0.03*	0.27±0.01	0.33±0.02	0.29±0.02	0.39±0.02*	0.36±0.01	0.43±0.02	0.41±0.01	0.48±0.03	0.44±0.02

Footnote table 2: Comparison of maternal care variables between LTIs (long tonic immobility) and STIs (short tonic immobility) females for the 5 observation days during the brooding period (mean ± SEM). Mann-Whitney U-test: *p<0.05, **p<0.01. Bold: highest values.

##### Vocal interactions with chicks

Maternal vocalizations differed significantly between females, confirming the trend we observed during the first hours spent with chicks. More STIs than LTIs cooed on PHD 2 (LTI: 3/22, STI: 11/21; Fisher exact probability test, p = 0.008) and on PHD3 (LTI: 1/22, STI: 7/21; Fisher exact probability test, p = 0.02), and more coos were emitted by STIs than by LTIs on these two days (table 2). On later days, the proportions of females that cooed and the numbers of coos did not differ significantly between the two sets (p>0.05).

##### Pecking, trampling and distances

On PHD3, 6 LTIs pecked chicks whereas only one STI did (Fisher exact probability test, p = 0.054): LTIs pecked more than did STIs (table 2) whereas on PHD5, 7 LTIs pecked chicks while only 2 STIs did, but neither differences in numbers of peckers (Fisher exact probability test, p = 0.08), nor frequencies of pecking were significant (Table 2). On other days, pecking frequencies did not differ significantly (p>0.05). Attacks on chicks were rare (only 5 aggressions in all were recorded during the whole brooding period, expressed by only 3 females) and consequently no differences between LTIs and STIs could be evidenced for any of the brooding days (p>0.05). When they were not being warmed, LTIs chicks were observed farther from their mothers on PHD2 and PHD5 (table 2). Trampling never differed significantly between LTI and STI (p>0.05).

##### Mothers’ activity

General activity levels did not differ significantly between LTIs and STIs (p>0.05) except on PHD3 when STI were more active (time spent in activity: LTI: 21.8±1.8%, STI: 26.9±1.7%, U = 147.5, p = 0.043). Nevertheless, females’ time budgets presented several differences: LTIs presented more alerts on PHD2 (LTI: 1.4±0.5%, STI: 0.2±0.2%, U = 167.5, p = 0.02), STIs spent more time eating on PHD3 (LTI: 10.7±1.0%, STI: 15.7±2.1%, U = 148.5, p = 0.05) and PHD5 (LTI: 9.7±1.1%, STI: 15.6±1.7%; U = 128.5, p = 0.01), whereas LTIs spent more time resting on PHD5 (LTI: 11.1±1.6%, 6.9±1.6%; U = 139, p = 0.03) and tended to rest more on PHD7 (LTI: 10.5±1.6%, STI:6.3±0.8%; U = 157.5, p = 0.07) and PHD 9 (LTI: 16.1±2.6%, STI: 10.9±2.9%; U = 153.5, p = 0.06). On PHD7, STIs spent more time pacing stereotypically along the cage walls (LTI: 1.8±0.9%, STI: 3.6±0.8%; U = 139.5, p = 0.02).

##### Responses to separation

After chicks were removed from their cages LTIs took longer to self-preen again (LTI: 258.9±14.7 s, STI: 178.9±25.2 s; U = 141, p = 0.02), and STIs moved (LTI: 0.982±0.269, STI: 1.552±0.209 per min.; U = 136.5, p = 0.02) and paced stereotypically (LTI: 0.100±0.052, STI: 0.343±0.104 per min.; U = 159, p = 0.04) more frequently. Females’ latencies to emit vocalizations (including both calls and coos), to feed and to rest did not differ significantly between sets (all p>0.05).

### Chicks’ Development

Chicks’ weights did not differ significantly between sets either immediately after separation from mother (LTI-c: 54.77±1.22 g, STI-c: 54.13±1.36 g; p>0.05) or one week later (LTI-c: 176.73±2.54 g, STI-c: 170.42±2.93 g; p>0.05).

#### Chicks’ fearfulness

Neither tonic immobility durations nor numbers of induction attempts differed significantly between LTI-cs and STI-cs (p>0.05).

In the emergence test, latencies to put their head out of the shelter or to emerge completely did not differ significantly between LTI-cs and STI-cs (p>0.05), but LTI-cs tended to wait longer between head and body emergence (LTI-c: 1.77±1.06 s, STI-c: 0.66±0.50 s; U = 1013, p = 0.06). After emergence, LTI-cs expressed more observations in low posture (LTI-c: 0.698±0.084, STI-c: 0.415±0.054 per min.; U = 628, p = 0.02), more freezing (LTI-c: 0.124±0.031, STI-c: 0.024±0.014 per min.; U = 675, p = 0.007) and more crouching fear postures (LTI-c: 0.093±0.028, STI-c: 0.024±0.014 per min.; U = 738, p = 0.04).

In the open-field, STI-cs expressed more observations in high postures (LTI-c: 0.131±0.059, STI-c: 0.341±0.081; U = 615, p = 0.03 per min.) and explored the floor of the apparatus more (LTI-c: 0.155±0.050, STI-c: 0.402±0.091 per min.; U = 664, p = 0.03). Four LTI-cs made flight attempts whereas no STI-cs did. This difference was not significant (Fisher exact probability test, p>0.05) but resulted in a higher number of flight attempts by LTI-c (LTI-c: 0.048±0.023, STI-c: 0.0±0.0 per min.; U = 779, p = 0.05).

Immediately after a startling sound was emitted, 5 LTI-cs reacted by freezing whereas no STI-cs did (Fisher exact probability test p = 0.03, table 3). The proportions of chicks that reacted by moving, observing, or that did not react, did not differ significantly between the two sets (p>0.05). During the two minutes following the emission of this sound, 6 LTI-cs expressed runs and 6 LTI-cs expressed fear postures, whereas no STI-cs expressed these behaviours (Fisher exact probability test: runs: p = 0.03; fear postures: p = 0.03) resulting in more runs and fear postures by LTI-cs (Table 3). The number of LTI-cs and STI-cs that expressed freezing or observations during this period did not differ significantly, but LTI-cs expressed more freezing than STI-cs, and STI-cs expressed more observations than LTI-cs (table 3).

**Table 3:** Immediate reactions of LTI-cs and STI-cs to a startling sound.

Parameters	LTI-c	STI-c
Immediate reaction		
Moves	8	10
Freezes	**5**†	0
Observation	27	29
No reaction	2	2
After startling stimulus		
Runs	0.071±0.032*	0
Freezes	0.321±0.053*	0.171±0.037
Fear posture	0.071±0.027*	0
Observation	1.048±0.109**	1.488±0.124

Footnote table 3: Number of subjects and mean (±SEM) frequencies of behaviours expressed by LTI-cs (chicks fostered by long tonic immobility females) and STI-cs (chicks fostered by short tonic-immobility females) during the 2 minutes following the emission of this sound. Fisher exact probability test: †p<0.05; Mann-Whitney U-test: *p<0.05, **p<0.01.

### Relationship between maternal behaviour and chicks’ fearfulness

Maternal care variables related to Japanese quail maternal styles [27] were averaged form PHD2 to PHD5. LTIs and STIs did not differ significantly for any variable related to the “rejection” dimension of care: proportions of time spent warming (LTI: 76.62±2.23%, STI: 78.25±1.39%; U = 215.5, p = 0.92), proportions of covering posture (LTI: 93.90±1.70, STI: 97.10±1.00%; U = 175, p = 0.25) and proportions of warming breaks initiated by the mother (LTI: 47.6±7.1%, STI: 50.40±4.5%; U = 218.5, p = 0.99). In contrast, variables related to the “aggressive” dimension differed significantly between the sets. On average LTI pecked daily more than did STI (LTI: 1.32±0.30, STI: 0.42±0.24; U = 141.5, p = 0.02) and stayed further from their chicks (distance index: LTI: 0.36±0.02, STI: 0.30±0.01; U = 133, p = 0.03). They also tended to be more aggressive (LTI: 0.30±0.18, STI: 0.00±0.00 per day; U = 190, p = 0.09). However, STI trampled their chicks more than did LTI during this period (LTI: 1.68±0.90, STI: 5.10±2.04; U = 140.5, p = 0.02). Behaviours related to the aggressive style of females were correlated with chicks’ fearfulness. The more aggressive the females behaved, the more reactive their chicks were during tests. Indeed, aggressions were positively correlated with freezing expressed by chicks in the emergence test (ρ = 0.325, p = 0.036). Similarly, mothers’ pecking was positively correlated with observations expressed in a low posture in the emergence test (ρ = 0.379, p = 0.014) as well as the expression of fear postures during the reaction to the startling sound (ρ = 0.491, p = 0.001). None of the other maternal behaviours related to maternal styles were not correlated with chicks’ fearfulness (p>0.05).

Other behaviours that differed significantly between LTIs and STIs were not found to be predictors of chicks’ subsequent fearfulness. Latency to become maternal and frequency of cooing (on PHD2 and PHD3) were not correlated with chicks’ behaviours (p>0.05). Similarly, warming breaks and proportions of covering postures on PHD9 were not correlated with chicks’ fearfulness variables (p>0.05).

## Discussion

This study evaluated how fearfulness of adult female Japanese quail influenced the way they care for foster chicks. We found that females’ fearfulness modified the rapidity of emergence of maternal responses after induction, maternal vocalizations, their physical interactions with chicks and their time budgets during the care period. After separation from their mothers, chicks brooded by LTIs were more fearful than chicks brooded by STIs.

### Maternal fearfulness

We confirmed first that LTIs and STIs’ TI responses differed; the differences found between females of the two lines were clear and strong, as TI was much easier to induce in LTIs and their TIs lasted much longer than did STIs’. Given the well-established strong differences between LTIs and STIs for other behavioural tests, we considered that the reactions of our females would also differ in novel environments, in the presence of a novel object or humans: LTIs would present more freezing in an open-field and longer emergence latencies in the emergence test [24], [29] and would express more fear behaviours in the presence of humans [24], [40] than would STIs.

### Fearfulness affects maternal behaviour

When opening the nest-boxes after the induction night, we found that this induction had caused no mortality either of LTIs’ or STIs’ chicks. As STIs are known to react actively when placed under such conditions [10], we feared they would inflict injuries on their chicks, but stimulations by chicks appear to have been sufficient to inhibit this reaction. Once broods and mothers were freed in their cages, STIs expressed maternal behaviour much faster than did LTIs who took nearly twice as long. This difference in maternal care emergence induced more signs of hypothermia in LTI-cs during the first hours following box opening. Moreover, during these first hours the females spent with their brood, vocal interactions with chicks differed significantly between LTIs and STIs. Indeed, fearful females never cooed whereas two-thirds of the STIs emitted this typical maternal vocalization. Later, during the first days of the brooding period, females’ physical and vocal interactions with chicks differed between the sets. LTIs pecked chicks more than did STIs and their vocal communication was still reduced as they cooed much less than did STIs. Simultaneously, LTIs appeared more anxious as they expressed more alerts. Furthermore, we found that, during the first days of the brooding period, LTIs were further from their chicks, probably because of their more fearful reactions to chicks’ solicitations (i.e. pecking) or because STIs cooed more, thus inducing the brood to rally round them. Both the presence of negative interactions and the limited vocal communication seem to impair spatial cohesion of LTIs’ broods, limited here by the physical constraints of the cage, but could lead to loss of chicks in more natural situations.

The present results indicate clearly that fearfulness can delay the emergence of Japanese quail’s maternal behaviour and impair the quality of their first maternal interactions with chicks. This is comparable to the effects previously reported for several mammalian species either in adoption procedures or natural bonding [18], [41]–[43]. Mammals’ fear reactions to offspring must be inhibited to enable the activation of maternal responses [44]. Similarly, differences in their fear of chicks could be at the root of the differences between the two sets of females. Initiation of mammals’ maternal care can be impaired by neophobia, particularly for a first brooding experience, as offspring constitute a novel stimulus [18]. The first interactions between Japanese quail and their chicks improve after a first maternal experience [35]. Nevertheless, studies investigating LTIs’ and STIs’ reactions to novelty report contradictory results [45]–[47]. The significant differences we found here concerning LTIs’ and STIs’ reactions after their brood had been freed in the cage suggest that chicks constitute a different kind of novel stimulus, able to reveal important differences in female’s reactions in a familiar environment. A previous study suggested that maternal fearfulness could predict maternal care as far as aggressiveness (one of the two dimensions of quail’s maternal styles) is concerned [27]. Using a similar method to calculate maternal care behaviour averaged over the 5 days post-hatch, our results confirmed this link. Indeed, LTIs expressed more aggressions, pecked more and were further from their chicks. These three behaviours are related to the “aggression” dimension of styles, whereas time spent warming, warming posture quality and contact breaks, related to the other dimension of care labelled “rejection”, were not influenced by maternal fearfulness.

The differences observed between the two sets decreased swiftly after the first days following sensitization. This suggests that LTIs habituate to chicks or that stimulations from chicks were able to improve LTI females’ poor maternal performance, in a way similar to the re-establishement of proper care by abusive or neglectful primate females following tactile stimulations by infants [48]. Nevertheless, some differences reappeared at the end of the brooding period, particularly concerning the way females expressed rejection of chicks. Rejection during the last part of brooding periods is necessary to promote dispersal of offspring [49]–[51]. Our results show that LTI and STI females express two very different strategies to reject chicks. LTIs expressed lying-on-one-side more frequently, a posture that prevents chicks from being warmed and that we consider as a form of passive rejection. Simultaneously, STI expressed a form of more active rejection by initiating more warming breaks. These two forms of rejection match LTIs’ and STIs’ global coping styles, characterized by LTIs’ greater behavioural inhibition [52], suggesting that the difference of fearfulness between LTIs and STIs affects maternal care until the end of the brooding period.

### Maternal behaviour affects chicks’ fearfulness

The differences between LTIs’ and STIs’ chicks we evidenced here clearly illustrate non-genomic transmission of maternal fearfulness, as chicks brooded by the more fearful LTIs were more fearful than chicks brooded by STIs. This result is consistent with previous studies on Japanese quail [24], [25] and confirms the influence of mothers on the development of birds’ fear responses similar to that reported for mammals [7]. The most parsimonious hypothesis obviously considers that the differences observed between chicks result from differences in maternal behaviour, or at least their mothers’ behaviour during the brooding period.

Maternal behaviour differed between LTIs and STIs mainly at the beginning and at the end of the brooding period, two stages when maternal behaviour is reported to have a large impact on offspring development [18], [53]. The quality of maternal responses to offsprings’ early solicitations is particularly important for the establishment and the quality of the mother-offspring bond in birds [54] as in mammals [55]–[57]. Our observations during the beginning of the brooding period indicate that LTIs and STIs did not respond similarly to chicks’ solicitations, solicitations that we consider to be similar initially, as all our chicks came from the same strain and were distributed randomly between the females of each set. Indeed, during the first hours following release of broods in the cages, LTIs took longer to start to brood their chicks, failed to communicate vocally with their broods and expressed aggressive behaviours like pecking. We suggest that, as for mammals, a mother’s reduced responsiveness can induce insecure attachment, leading to increased responsiveness to stressful events [58], [59].

LTI and STI females also clearly differed in the way they promoted dispersal at the end of the brooding period. The patterns of brooding period ending are particularly important for the behavioural development of offspring. Gradual and brutal weaning of altricial rodents have different consequences on the emotional reactivity of offspring [53], [60]. Our results suggest that the pattern of dispersal promotion could have the same consequences on the emotional development of quail offspring as the pattern of weaning. Indeed, STIs actively rejected chicks before they reached the age at which they would naturally emancipate whereas LTIs expressed more passive warming refusals and consequently separation between LTI-c and their mothers on PHD 10 was more abrupt and could have induced higher fearfulness.

Correlation data seem to support the first of these two hypotheses. Our results confirm the link between early maternal behaviour and chicks’ fearfulness. More particularly, during this precocious period, maternal aggressiveness seems to be the most important component of maternal care involved in the transmission of LTIs’ and STIs’ fearfulness to their respective chicks. This result stresses the importance of early adverse events on the development of stress reactivity, well documented for both human and animal models [see 57 for review]. Neither latency to become maternal nor the way females express rejection during the late brooding period appear to be responsible of differences observed between LTI-Cs and STI-Cs.

In addition to these mechanisms, the fact that precocial birds are capable of learning as soon as they hatch [32] and even before hatching [61], suggests that active learning mechanisms could be implied. Young mammals can learn fear reactions by observing adult models [62]–[64]. LTIs were more alert, less mobile and globally less active during our observations and they are also known to be more fearful of humans [24], implying that they reacted differently to interventions by care-givers in the presence of their chicks. We suggest that chicks could also have learnt a pattern of responses to stressful events by observing their mothers during the brooding period.

## Conclusion

Our findings demonstrate that fearfulness affects the expression of birds’ maternal care. Fearful females show impaired maternal behaviour characterized by a reduced vocal communication with chicks, aggressive interactions on the first days of brooding and incapacity to actively promote chicks’ dispersal. Although our favourable environmental conditions thwarted mortality, all our evidence suggests that this impaired maternal care by fearful females would have deeper consequences on the growth and survival of chicks in more natural situations, as for mammals [17], [65].

Our results also indicate strong similarities between the consequences of mammals’ and birds’ fearfulness on maternal care and, more widely, on the relationship between temperament and maternal behaviour. As for mammals, maternal care seems to be the bridge between mothers’ and chicks’ fearfulness, probably associated with active learning of fear reactions by the highly precocial quail chicks. Biochemical mechanisms underlying the influence of maternal care on chicks’ emotional development should now be explored. Notably, differences in quality of the physical interactions between mothers and chicks suggest a possible implication of epigenetic mechanisms similar to those reported for altricial rodents [2], [66], [67]. Modifications of genome expression in response to the environment have previously been reported for precocial birds [68], but never associated with maternal care.

Finally, our results raise the question of the long-term and cross-generational consequences of maternal behaviour. Future studies will have to use longitudinal procedures to describe the consequences of maternal fearfulness on adult offspring behaviour, notably on parental care responsible for potential transmission to subsequent generations.
